# The Multi-Parameter Wireless Sensing System (MPwise): Its Description and Application to Earthquake Risk Mitigation

**DOI:** 10.3390/s17102400

**Published:** 2017-10-20

**Authors:** Tobias Boxberger, Kevin Fleming, Massimiliano Pittore, Stefano Parolai, Marco Pilz, Stefan Mikulla

**Affiliations:** 1 Helmholtz Center Potsdam—GFZ German Research Centre for Geosciences, Helmholtzstrasse 7, 14467 Potsdam, Germany; kevin@gfz-potsdam.de (K.F.); pittore@gfz-potsdam.de (M.P.); parolai@gfz-potsdam.de (S.P.); marco.pilz@gfz-potsdam.de (M.P.); stefan.mikulla@gfz-potsdam.de (S.M.); 2 Istituto Nazionale di Oceanografia e di Geofisica Sperimentale—OGS, Borgo Grotta Gigante 42/C, 34010 Sgonico (TS), Italy; sparolai@inogs.it

**Keywords:** natural hazard and risk, EEW, multi-hazard/risk, rapid response, monitoring

## Abstract

The Multi-Parameter Wireless Sensing (MPwise) system is an innovative instrumental design that allows different sensor types to be combined with relatively high-performance computing and communications components. These units, which incorporate off-the-shelf components, can undertake complex information integration and processing tasks at the individual unit or node level (when used in a network), allowing the establishment of networks that are linked by advanced, robust and rapid communications routing and network topologies. The system (and its predecessors) was originally designed for earthquake risk mitigation, including earthquake early warning (EEW), rapid response actions, structural health monitoring, and site-effect characterization. For EEW, MPwise units are capable of on-site, decentralized, independent analysis of the recorded ground motion and based on this, may issue an appropriate warning, either by the unit itself or transmitted throughout a network by dedicated alarming procedures. The multi-sensor capabilities of the system allow it to be instrumented with standard strong- and weak-motion sensors, broadband sensors, MEMS (namely accelerometers), cameras, temperature and humidity sensors, and GNSS receivers. In this work, the MPwise hardware, software and communications schema are described, as well as an overview of its possible applications. While focusing on earthquake risk mitigation actions, the aim in the future is to expand its capabilities towards a more multi-hazard and risk mitigation role. Overall, MPwise offers considerable flexibility and has great potential in contributing to natural hazard risk mitigation.

## 1. Introduction

The paramount requirement for effective disaster mitigation, preparation and response is the provision of relevant, timely, accurate and adequately spatially-resolved information. Such information will need to cover both the natural hazard environment (e.g., an area’s climate, seismicity, landscape, etc.), possible contributions from, e.g., heavy industry (e.g., potential for toxic material release), and the nature and extent of the area of interest’s built environment. However, this leads to the problems of, first, identifying what parameters need to be measured, second, how to interpret and communicate these measurements, and third, what is the most effective (and cost efficient) means of undertaking these data-gathering actions (including the establishment of the required measuring networks). This is especially important in those regions where there is a lack of such information, or where there are major changes occurring in the environment, e.g., rapid urbanization in developing countries, where available information may quickly become out-of-date. In addition, those environments that experience a number of different natural hazard types (e.g., earthquakes, landslides, windstorms, flooding) would require multiple parameters to be measured.

The aim of this paper is to present an integrated system for measuring a range of parameters that are relevant to different components of the so-called risk equation. Risk in general may be described as being a function of hazard (e.g., earthquake-induced ground motion), exposure (e.g., the number and structural characteristics of buildings within a given area, but also segments of the population, lifelines or the natural environment) and the vulnerability of the exposed elements to the considered hazard. Knowledge of each of these elements calls upon specific types of information. For example, understanding the hazard requires records of the frequency of events of a given size (e.g., earthquake magnitude, flood water levels), exposure requires the extent and distribution of the elements of interest, be it population density (and the associated demographics), industrial facilities and lifelines and other infrastructure, while vulnerability describes how each element is affected by an event of a given size. The role of the system presented in this work is to undertake measurements either continuously, as in the case of structural health monitoring and early warning, or for specific tasks, e.g., local site amplification assessments, monitoring following a seismic event, that will contribute to the disaster mitigation and response decision-making process.

An earlier generation of smart sensors, termed Self-Organizing Seismic Early Warning Information Network (SOSEWIN, [1]) were developed by GFZ in collaboration with the Humboldt University within the framework of the EC-funded Seismic eArly warning For EuRope project (SAFER; http://cordis.europa.eu/project/rcn/81386_en.html). Based on this experience, a new generation of smart sensors have been designed that extend the capabilities of the SOSEWIN technology into an industry-grade solution. The Multi-Parameter Wireless Sensing system (MPwise, [2]) presented in this work will contribute to the provision of some of the required information, focusing on the hazard and vulnerability components of the risk equation. The system allows several types of sensors to be combined with the node unit to acquire data in real time and, when combined with the software platform, is capable of real-time processing. While such a system could be used for long-term risk assessment, it may also be employed to obtain knowledge about an evolving situation, for example, the change in the fragility of buildings due to damage arising from a seismic sequence. Another possible use is for early warning where, for example, in the case of earthquakes, it may detect and undertake preliminary processing of recorded ground motion. This may include predicting damage to structures, as well as the activation of an appropriate alarm system (e.g., audio-visual), or the sending of messages to designated stake holders (e.g., civil protection, infrastructure operators). While the focus of the system’s development has so far has been on seismic hazard and risk, the long-term goal of the work is on a multi-hazard system. Another important criteria considered during its development is its ease of use/installation. This is to enable its exploitation (and that of the accompanying processing software) by Civil Protection (CP) authorities with the minimal amount of training and without the need for specialized expertise.

The next section provides a general overview of the MPwise, including a detailed description of its central hardware, communications protocols, current processing capacity and possible applications. This is followed by a description of possible applications, including -site and regional earthquake early warning (EEW) systems (Section 3), structural monitoring characterization (Section 4) and site amplification assessments (Section 5). The paper concludes (Section 6) with a summary of the ongoing development and the outlook for additional ways the system may be exploited. Note that much of the work in this paper was undertaken within the context of the SeIsmic monitoring and vulneraBilitY framework for civiL protection project (SIBYL; www.sibyl-project.eu). Hence, the figures showing output from the developed system will have the SIBYL logo attached to it. 

## 2. The MPwise System

### 2.1. General

The MPwise is an advancement from previous activities that set out to develop a wireless system for EEW and structural monitoring (e.g., [1,3,4]). One of the primary ideas behind this work was for the sensing units or nodes (when employed in a network) to be “low cost”. That is, they are made up of off-the-shelf components, whereby they may be deployed in much denser networks than is possible with conventional seismic sensing equipment. Another of the concepts behind the development of this system was to shift part of the processing burden from a centralized location to the nodes themselves, connected via a wireless mesh network. There are several advantages of such a configuration:

- The resulting system would be more resilient to external disturbances,
- Useful information could be already available at the node level for undertaking automatic (unsupervised) emergency measures,
- By processing data at the node level, potentially no raw-data needs to be transmitted across the network, freeing it for the circulation of only the resulting (higher-level) information while allowing a narrower communication band and prompter response of the system.

The resulting network would be able to determine the optimal wireless communication path which would also be able to automatically adapt itself given changing circumstances (e.g., nodes becoming inoperable, interference, etc.).

**Figure 1:**
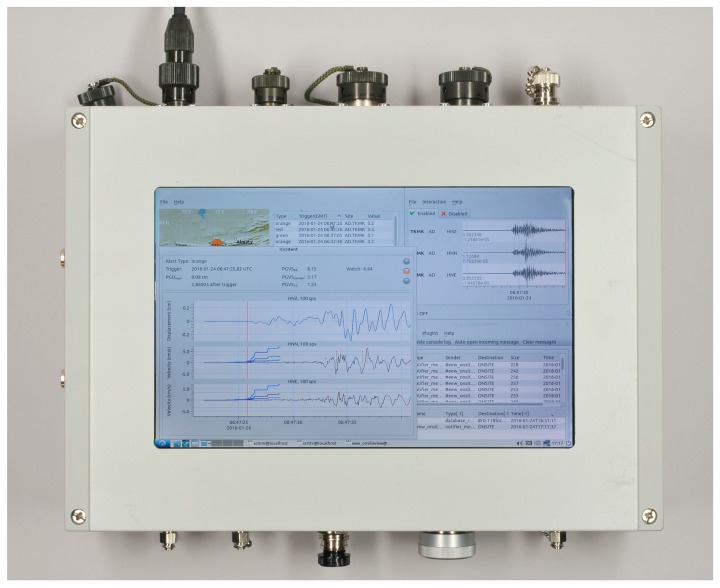
A MPwise unit. This version includes a touchscreen embedded in the housing. While this arrangement is not standard, an external touchscreen can be connected to all units. In this image, the touchscreen display shows the operation of the onsite EEW software described in [5,6,7]. The dimensions of the latest version are: length 20.5 cm, width 16 cm, and height 8 cm.

Since these initial stages, the units have advanced in step with the increasing capacity of off-the-shelf components, while ideas about how such a system may be exploited have also developed. Figure 1 shows the latest version of the MPwise, including an example of how its internal software may be used and displayed on the unit’s screen (in this case for on-site early warning), while Table 1 lists examples of its possible use, such as pre- and post-event building monitoring, site amplification assessments and on-site EEW. Table 2 in turn describes how the different roles that may be played by MPwise units and networks will involve their various components (sensing, communications and processing). Details of the hardware, software, communications and processing characteristics of the current system are provided in the following sections.

### 2.2. Hardware

**Figure 2:**
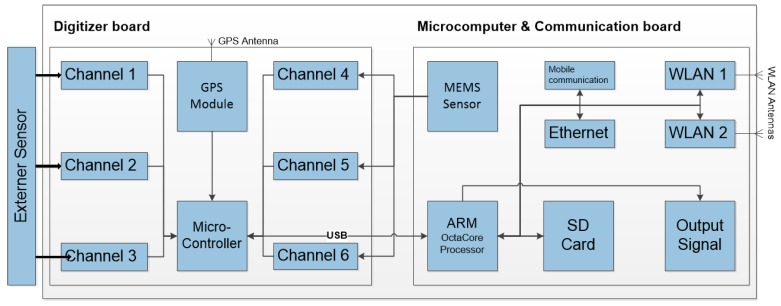
General schema of the MPwise units, showing the three main components: the digitizer board (see Table 3), the microcomputer (which includes the communication periphery, see Table 4) and the links to the internal and external sensors.

The system is designed in a modular manner, hence allowing different configurations of sensors and communication interfaces to be employed, depending on the case at hand. Such an arrangement would also help in future developments when the system is expanded from seismic to other hazard types. The current MPwise hardware (Figure 1) consists of three main parts, as shown in Figure 2, which presents a schematic overview of the system’s architecture, namely the digitizer board (see Table 3), the microcomputer and communications board (see Table 4), and the external sensors.

- The digitizer board (Table 3), i.e., an analogue-to-digital converter (ADC), was designed as a low-power consumption solution and considered special seismic requirements such as a high resolution (number of bits) in the analogue to digital conversion, exact time marks (using a (Global Navigation Satellite System—GNSS—module which is also used for locational information) and minimal drift in the time accuracy included in its design. The board is equipped with a 6-channel ADC which has a resolution of 24 bits, with the sampling rate selectable from 50 to 400 samples per second (sps).
- The microcomputer (Table 4) board has three roles: (1) acquisition and storage of data; (2) data processing and analysis; and (3) communications, i.e., it includes the periphery for data transmission. It is made up of an embedded 8-core ARM processor (http://www.arm.com/; see Table 4) that uses a micro SD card (currently 32 GBytes, but easily expandable) as a local data storage device. The communications hardware linked to the microcomputer board consists of two omni-directional dual-band antennas for WLAN communication and one omni-directional antenna for mobile communications.
- The sensors themselves (see Table 2). Each unit includes a high performance 3-axis ultracompact analogue accelerometer, based on Micro Electro Mechanical Systems (MEMS), which have been successfully employed in various seismic networks (e.g., [1,3,4]) as well as for field acquisition by the exploration sector [13]. Different external sensors can be further connected to up to six input channels. Tested configurations include standard strong and weak motion sensors, broadband sensors, and USB-connected devices such as video cameras, temperature and humidity sensors, and GNSS systems (note, the latter is different from the internal GNSS used for the time synchronization).

Most of the components are off-the-shelf, with the exception of the digitizer board, which has been developed by GFZ Potsdam (http://www.omnirecs.de/).

### 2.3. System Software

The main system software operating on the nodes consists of the following:

- The operating system is based on a Linux kernel and GNU software, which is open-source, freely available and highly configurable. By default, it contains only the minimum that is required to run Linux without a desktop environment.
- SeisComP: The Seismological Communication Processor (SeisComP; Seiscomp3, https://www.seiscomp3.org/) is an open-source software package and concept for real-time seismic data acquisition, processing (quality control, event detection and location), distribution and interactive analysis developed by the GFZ. It includes the SeedLink protocol (http://www.seiscomp3.org/wiki/doc/applications/seedlink), which is the system devoted to the real-time seismic data distribution, that is, a server protocol based on the Transmission Control Protocol (TCP; TCP/IP, https://en.wikipedia.org/wiki/Transmission_Control_Protocol). There is also software embedded within this package for on-site EEW.
- Cube_plugin: This program is embedded in SeisComP and handles the data streams from the digitizer board, and then archives them via a SeedLink (see below) or common acquisition protocol (CAPS) server (this has been developed by gempa GmbH; http://www.gempa.de/caps/). One of the advantages of CAPS over SeedLink is the greater ease with which one can transfer multi-sensor data from the node to a gateway node, which in turn transmits the information to a data center or end user. For example, this feature allows the optimization of the joint transfer of seismic data and images using a unified protocol.
- Optimized Link State Routing (OLSR Project): OLSR (OLSR, http://www.olsr.org) is a table-driven proactive routing protocol currently chosen for the wireless mesh network. As a proactive protocol, it periodically assesses and maintains the network topology by sharing information about its direct neighborhood throughout the network. OLSR is capable of operating with hundreds of nodes, and it is also widely accepted by several mesh networking communities (FREIFUNK, https://freifunk.net/).

### 2.4. Communications

The data transmission follows the real-time approach already developed in the SOSEWIN and is dependent on either single station or network topologies. The communications can be managed via LAN, mobile communication (GSM, UMTS, LTE (mobile broadband, https://en.wikipedia.org/wiki/Mobile_broadband)), or a self-organizing wireless mesh network. The most important reason for having a self-organizing network is to allow it the flexibility to adapt to dynamic environments which may lead to variable numbers of sensors. This can occur when, for example, a significant event occurs (e.g., earthquake) where several nodes of the system might be disabled due to damage or power outage. The system can therefore find alternative routing among the remaining nodes to restore a fully operational communications network.

The communication of the results of the processing at each node throughout the network is based on the routing concept. The term routing refers to the procedure of selecting within a network the paths along which data can be sent from a source to a sink. Routing activities within a wireless network are made more complicated by the fact that all nodes act contemporarily as senders, transmitters and receivers of data. The main advantages of wireless mesh networks for seismic networks or arrays within urban environments are (1) the system is free from cable usage, thus, allowing improved array geometries and azimuthal coverage; and (2) in the case of large arrays, data can also be transferred to a user by multi-hop communications from those instruments that are not in a line-of-sight with the user itself, or are too distant (remote stations). As mentioned above, the OLSR that the MPwise employs is a proactive routing protocol, where every node has a map (routing table) of the complete network topology, allowing data to be immediately sent along the optimal path towards the users or gateways. It makes use of advanced metrics, i.e., measurement methods, for evaluating a multi-hop path within the network. The two wireless modules integrated in the MPwise system are following the 802.11 bgn wireless standard with a data rate for 802.11 n up to 150 Mbps. For this reason, no problems related to the data communication are expected for our applications (small size wireless arrays and building/structural health monitoring). In the past, various station configurations have been tested using up to 30 sensors simultaneously and exploiting real-time data communication without any problems. 

### 2.5. Embedded and Joint Processing on Node Level

**Figure 3:**
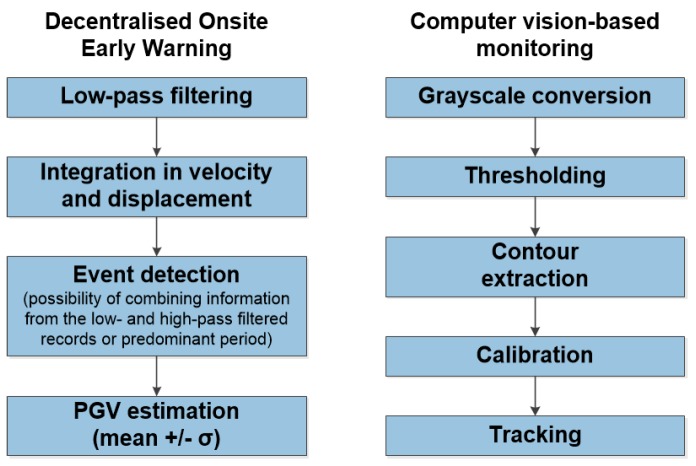
The steps followed when the MPwise units are being employed for (**a**) decentralized on-site earthquake early warning [5] and (**b**) computer vision-based monitoring roles. Note the GFZ-Sentry application would be employed for the decentralized on-site early warning.

Different methods of analysis are under development and implementation, dependent upon the response time required by the application (e.g., real-time for early warning, near-real-time for rapid response), either using directly the capabilities of each single node that constitutes the self-organizing network, or in a more centralized early warning center. The processing steps at the node level are being designed to be suitable for on-site early warning and the assessment of the predicted damage of buildings, computer vision-based monitoring, displacement estimation by joint processing of single-frequency GNSS and MEMS-accelerometer data, and the running of an automatic, audio-visual alarming system (which would involve using either the embedded or external touchscreen display, external loudspeakers and/or other external devices). For example, Figure 3 (left), shows the processing steps for one of the EEW options, termed decentralized on-site early warning [5,6,7], and for the computer vision-based monitoring application (see Section 6). With regards to other sensor types that may be attached to the MPwise, an example could be the use of external GNSS units (see [14]), for example, the u-blox GPS module (https://www.u-blox.com/de), which may be used in conjunction with the double-integrated acceleration data recorded by the internal MEMS (note, this GNSS module is in addition to the one used for the time stamping). In the following sections, we will present some examples of the different applications of the MPwise. One of the software tools installed on the MPwise is the GFZ-Sentry (GFZ-Sentry is being developed by GFZ in collaboration with gempa GmbH). This is a software application that runs in real-time and analyses the recorded ground motion for on-site and decentralized early warning. The software, which has been tested extensively on off-line recordings [7] exploits the first three-seconds of a triggered signal to determine (making use of empirical relationships) what the potential ground shaking at a site will be, which is then used to make a decision as to whether to issue an alarm (and of what type) [5,6,7].

## 3. Applications: Earthquake Early Warning in Kyrgyzstan

**Figure 4:**
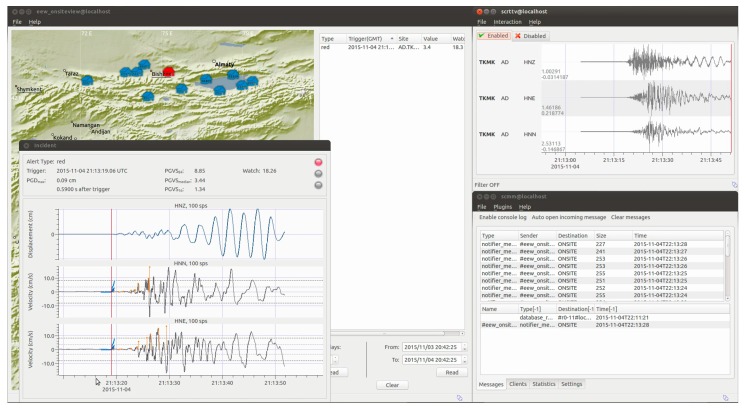
Example of output from the Seiscomp3 system implemented within the EEW software module of the MPwise, based on the GFZ-Sentry software developed at the GFZ [5,6,7] for the case of the Kyrgyz Republic. Note that this processing and associated images may be produced by a data center computer connected to the recording network, or on the touchscreen embedded in or connected to one of the units themselves (see Figure 1).

Earthquake early warning systems (EEWSs) in their various forms are recognized as potentially valuable tools for mitigating against the risk associated with earthquakes. Until recently, most on-site EEWS have attempted the timely estimation of the incoming danger either through a rapid (and first order) estimation of the magnitude and location of the event, and then estimating the possible ground motion that the site will experience [15,16], or by directly estimating the ground motion depending on the peak values of the ground displacement measured during the first few seconds of the first arriving P-waves (e.g., [17,18]). During the last decade, in order to increase the efficiency of EEWSs, an end-to-end approach where the concept of early warning was coupled with the expected structural performance has been under development (e.g., [19]). Following this approach, early warning, structural analysis, and damage and loss analyses are combined within a performance-based framework upon which a decision-making procedure can be established (e.g., [20,21]). Exploiting the computational power of modern sensing units, such as those used in the MPwise units, the implementation of this concept can be transferred to each unit, following a decentralized performance-based early warning scheme (see [6] Figure 4).

The deployed instruments would thus be able to serve a number of functions, such as

- Implementing a threshold-based on-site early warning system (OSEWS) that can also be employed following a decentralized approach for specific infrastructure;
- Monitoring the structural response, and its changes, of buildings and infrastructure in real time during an after-shock sequence;
- Assessing the expected damage to nearby structures soon after an event’s occurrence;
- Estimating the overall expected damage to a target structure during the aftershock sequence;
- Validating damage forecasts determined by, e.g., probabilistic approaches, and updating the fragility curves based on recorded ground motion.

The value of having the capacity for onsite processing is that for a particular site, a detected event may be analyzed and, if necessary, a warning issued more rapidly, than would be possible if the recordings had to be transmitted to a centralized processing center.

## 4. Application: Building Measurements and Structural Monitoring in Thessaloniki, Greece

**Figure 5:**
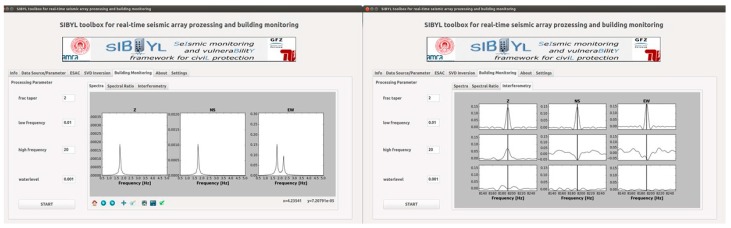
Example of output for short-time building measurements (note, this is the Graphical User Interface (GUI) developed for the SIBYL project for the case of the AHEPA hospital in Thessaloniki, Greece. The results may be viewed either on an embedded or external touchscreen). (**a**) Spectra computation to identify the resonance frequencies of the building. (**b**) Interferometry plot to evaluate the shear wave velocity inside the building.

As shown within the framework of the Strategies and Tools for Real Time EArthquake RisK ReducTion (REAKT; http://www.reaktproject.eu/) project, the combination of systems like the MPwise with permanent accelerometric arrays, like that which operates (since May 2012) in the reinforced concrete AHEPA hospital building in Thessaloniki, Greece (this hospital includes two 7-storey blocks, which are joined, but not strongly mechanically coupled, leading to each block responding almost independently of each other to ground motion), as well as temporary networks, makes it possible to evaluate the risk to structures under various earthquake scenarios. For example, measurements of ambient noise data made over even short periods of time may be used to evaluate the dynamic characteristics of a building, namely the natural frequencies (see Figure 5) and mode shapes, making use of such methods as system identification and Operational Modal Analysis, which may be performed by applying both nonparametric and parametric methodologies (e.g., frequency domain decomposition [22] stochastic subspace identification, [23]).

Long-term seismic noise measurements (from both natural and anthropogenic sources) and earthquakes recorded within buildings may be used to develop structural health monitoring techniques to identify a building’s structural conditions and fragility using both waveform and vibrational approaches [22]. This can provide, for example, a building’s mode shapes, which may be updated and better constrained over time as more observations become available, or when changes in the building’s structural behavior becomes apparent as a result of, for example, aging or minor damage arising from seismic events. It also allows predictions of how a building will respond (in terms of potential damage) given a level of ground shaking. The making of such recordings are one of the specific roles that the MPwise units have been designed for.

**Figure 6:**
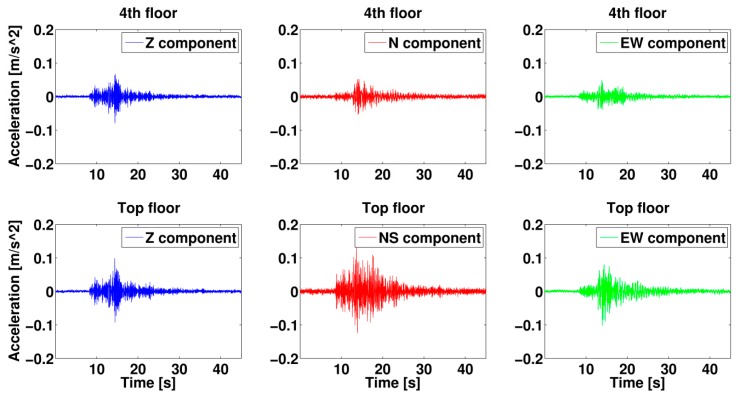
Earthquake data (11. October 2013 Volvi earthquake) for the east-west (EW), north-south (NS) and vertical (V) components recorded using MEMS sensors installed in a predecessor of the MPwise units (SOSEWIN, see [1]) deployed in the AHEPA hospital at the 4th floor and the roof.

Furthermore, a network made up of MPwise units may be used to develop an operational framework for EEW and rapid post-earthquake damage assessment. For example, Figure 6 shows earthquake data from the 11. October 2013 M4.5 Volvi event (http://geofon.gfz-potsdam.de/eqinfo/event.php?id=gfz2013txlu), recorded by the strong motion sensors deployed in the AHEPA hospital on the 4th floor and the roof. Such recordings, while potentially useful for structural measurements and EEW purposes, may also be used to investigate other earthquake-engineering issues, such as soil-building interactions (see [24]).

## 5. Application: Site Amplification Assessments in L’Aquila

Seismic noise is a highly variable and irregular assemblages of seismic waves (e.g., body waves, surface waves, and their related scattered and diffracted phases). Among them, surface waves (i.e., Rayleigh and Love waves) are considered to be the dominant and most coherent component. From these recordings, consistent and reliable estimates of parameters related to site effects may be retrieved from the analysis of the horizontal-to-vertical (H/V) spectral ratio curve [25] and the Rayleigh wave dispersion curves (e.g., [26,27]). The great interest that seismologists reserve for these waves is mainly justified by the relationship existing between their velocity and the subsoil structure, and, in particular, the S-wave velocity. Hence, considering that Rayleigh waves sample portions of the subsoil at depths proportional to their wavelengths, and that their phase velocities are strongly conditioned by the S-wave velocities of the layers sampled, they are used to deduce information about the subsoil structure, and in particular the local S-wave velocity profile and the so-called V_s30_ (average S-wave velocity in the upper-most 30 m). The local S-wave velocity profile may be found using the Single Value Decomposition (SVD) inversion technique applied to the Rayleigh wave dispersion curves [28]. The H/V curve is obtained by single-station seismic noise recordings, while the V_S30_ is estimated by the effective Rayleigh wave phase velocity corresponding to a wavelength of 40 m.

**Figure 7:**
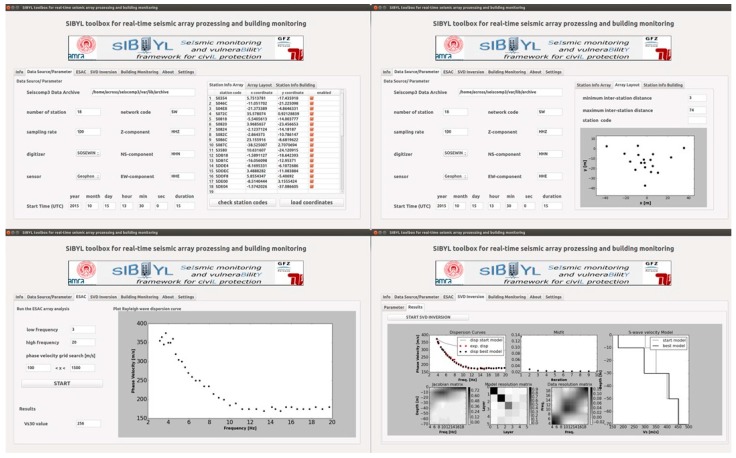
Output from the MPwise processing of seismic array data for site assessment (note, this is the Graphical User Interface (GUI) developed for the SIBYL project). Top left) The data source and parameter menu allows the user to configure different parameters, such as the number of stations, network code, sampling rate, channel identifier, digitizer and sensor specific constants, etc. Top right) Overview of the array geometry. Bottom left) Real-time processing of the array measurements using the extended spatial autocorrelation (ESAC) method [29] to obtain a Rayleigh wave dispersion curve. Bottom right) SVD (Single Value Decomposition) inversion to obtain a 1D shear wave velocity model, as well as a quality control provided by Jacobian, model and data resolution matrices.

The MPwise system is suitable for performing real-time analyses of seismic noise data gathered during seismic array measurements for site assessments. Once the system is deployed in the field, a relatively simple operation where the nodes, equipped with geophones, are distributed across an area (50–100 m across) with varying distances between nodes (3–65 m), the MPwise units create a wireless mesh network, allowing an operator to retrieve in real-time the data from all stations of the network, and to perform the analysis already in the field during the acquisition phase (see Figure 7). Each station in addition continuously and independently stores the data in miniseed (http://ds.iris.edu/ds/nodes/dmc/data/formats/miniseed/) format on its SD card.

## 6. Ongoing Experimental Applications: Computer Vision-Based Monitoring

There are also cases where the use of accelerometric sensors is not always optimal for structural monitoring. In particular, when the monitoring of displacement (or velocity) is needed, the process of double integration of the signal from the accelerometric sensors is biased by the baseline offset of the digital recording and often produces unrealistic results. The origin of such offsets is diverse and can be classified in terms of errors due to instrumental instability (non-linear instrumental response, limited resolution of the measuring system, insufficient sampling rate, level of electronic noise, etc.), background noise (depending on the target site), the estimation of the real initial acceleration, velocity and displacement values, and data manipulation. Alternative measurement techniques for displacement include, for instance, analog or digital strain sensors, which offer good performance, but over a reduced working range.

Several laboratory-based experimental set-ups (e.g., [30,31]) have demonstrated the potential of image-based monitoring. Displacement measurements of high-rise buildings based on conventional imaging have been proposed by [32,33], the latter using high-speed linear cameras, while [34,35] proposed the dynamic monitoring of slender structures using various computer-vision techniques. Most of these approaches involve 1-D or 2-D measurements, but with more complex arrangements, 3-D structural monitoring is also possible, as showed by [36,37,38]. The mentioned techniques and approaches can be used to integrate the pre-event assessment of fragility for residential structures, and to collect in real-time information about the health state of the structure during and after an earthquake (see also [32] for a specific application to damage detection). Furthermore, the prompt observation of, for example, overthrown furniture in the building or significant internal structural and non-structural damage would, in many cases, assist search and rescue operations in the aftermath of a damaging event.

**Figure 8:**
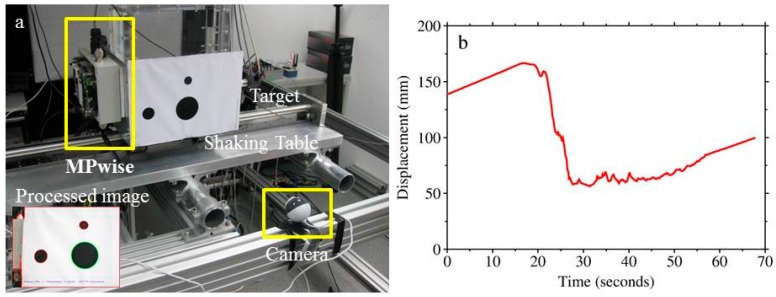
(**a**) The experimental set-up for the displacement measurements using a camera connected to a MPwise unit; (**b**) The displacement of the target point (red cross in the processed image in (**a**) as determined from the camera images.

Several laboratory tests of a prototype displacement measurement set-up using MPwise units connected to cameras have been carried out (see Figure 8). The experiment entails a visual target being fixed to a 1-D shaking table simulating realistic ground shaking. A low-cost USB camera, connected to the MPwise device, tracks in real time the position of the visual target and provides a repeated (at 5 frames per second) estimate of the observed target’s displacement.

The software work-flow for this application is depicted in Figure 3 (right). While the scale of the experiment is not representative of actual applications, the designed laboratory test allowed us to evaluate the feasibility of the ongoing efforts. The next step will include repeating this test with a much higher frame rate of about 90 frames/s, and extending it to real cases, such as its installation in buildings and other structures. Two approaches will be tested; for the first approach, the camera and target will be inside the building, the camera mounted on the floor and the target on the ceiling to provide information about the inter-storey drift. In the second, the camera is outside of the building and the target on top of the building to provide information about the absolute displacement of the building. In this case, the camera is not affected by the shaking of the building itself. Applications at larger scales (e.g., longer camera-to-target distances) mainly imply the selection of different hardware components (e.g., cameras with different focal lengths or resolutions), without a strong impact on the software solutions.

## 7. Conclusions 

The MPwise system, as seen by the range of applications outlined in this work, shows a great potential for a variety of seismic mitigation activities, including earthquake early warning, post-event aftershock recording, structural monitoring under various circumstances, and site assessment. Most of the components are off-the-shelf, thus reducing the cost of these units, leading them to be much less expensive than standard seismometers and digitizers, and therefore allowing more extensive and/or dense networks to be established.

One of the planned next steps is to expand the capacity of the system to include single-frequency GNSS receivers that are suitable for dense deployments because of their small size, low cost, and relatively low power consumption. Displacement monitoring systems using such instruments have been investigated in a number of studies. For example, [32] employed a low-cost L1 GNSS for static displacement measurements, in particular using the carrier phase signal. Another step will be including displacement measurements from the real-time analysis of acquired images, which will require moving from laboratory tests, as discussed in the previous section, to the establishment of networks that include camera sensors in actual structures.

In conclusion, the MPwise is a system that offers a range of possibilities for seismic, and eventually, multi-, hazard and risk assessment. Due to its ease of use and intended modular, flexible nature it offers great potential for cost effective risk assessment, for both pre- and post-event cases.

## Figures and Tables

**Table 1:** Examples of how the MPwise system could be employed.

	Examples/Cases of Its Use	Description
**Pre-event**	Pre-event building and structural monitoring.	The system can be installed in residential buildings and critical facilities. It exploits ambient seismic noise and micro-earthquakes to characterize the seismic response of the monitored structure in order to constrain or update its structural fragility model [8].
	Pre-event landslide monitoring and early warning.	Landslide potential can be assessed using a network of nodes equipped with high precision GNSS and seismic sensors. The system can be used to obtain further data on the soil structure (by 3D tomographic analysis of the target area) and to detect and monitor mass movements [9]. The resulting information would then be sent to the appropriate users while warnings may be issued and other emergency measures supported.
	Site amplification assessments	Information on the subsoil structure, and in particular the local S-wave velocity profile and the so-called V_s30_ can be extracted from seismic noise array measurements [3].
**During event, post-event**	On-site earthquake early warning.	The system detects the first ground motion on-site and estimates the potential maximum shaking at the node level (e.g., at critical facilities such as hospitals, industrial plants, gas pipelines, etc.), allowing local emergency measures to be automatically conducted for loss mitigation [5,6,7].
	On-site earthquake damage forecasting.	The system can be installed in order to cover one or more buildings (one or several nodes for each building). A structural fragility curve for the monitored building (or an approximate model) is embedded at the node level, leading to the ability to forecast the extent of damage to the building, starting from the early shaking. If the system forecasts severe structural damage, a warning is issued and emergency measures can be undertaken [5,6].
	Emergency communications.	The flexibility of the system can be exploited following the occurrence of an event to set up local emergency voice and data communication networks, both to connect different critical facilities and to support the operations of civil protection authorities [10].
	Earthquake post-event building tagging.	The damage that the system forecasts during shaking can be detected by comparing the frequency response of the structures before and after the event, or by using the on-board camera to detect a permanent drift of critical load-bearing structures. The system may then provide a preliminary tagging of the monitored building to facilitate civil protection operations. For instance, a building could be automatically issued a “red tag” if structural damage is detected, with a signal/alarm sent to the occupants warning them to leave the building, while also issuing a “priority for inspection” warning to reconnaissance personnel [11].
	Flood monitoring.	The system, when equipped with water-level measuring sensors, can monitor the evolution of a flood over both local and regional scales. An in-situ network could be deployed rapidly during the first onset of a flood, and be used to validate stochastic inundation scenarios and provide ground-truth for wide-scale assessments based on remote sensing approaches [12].

**Table 2:** The various possible applications of the data acquisition, communications and processing components of the MPwise.

	Components	Regional and On-Site (i.e., Decentralized) Earthquake Early Warning	Rapid Response Support	Building and Structural Health Monitoring	Site-Effect Estimation (Use of Two Dimensional Arrays)	Other Applications
**Acquisition**	External sensors (Broadband, Strong Motion, or Geophone)	√	√	√	√	Landslide monitoring
	Internal MEMS sensor	√	√	√		Landslide monitoring
	Full HD camera			√		Landslide, water levels monitoring
	GNSS receiver	√		√		Landslide monitoring
	Temperature and humidity sensors					Meteorological observations
**Communication**	LAN	√		√		Response to any other hazardous events
	WLAN including self-organizing wireless mesh network topology	√	√	√	√	Response to any other hazardous event
	Mobile communication (UMTS, LTE)	√	√			Response to any other hazardous event
**Processing**	Node level	√	√	√		Other hazardous events (landslides, floods)
	Network level	√	√	√	√	Other hazardous events (landslides, floods)

**Table 3:** Technical specifications of the digitizer board.

Number of channels	3 or 6
AD converter resolution/effective resolution	24 bit, typ. 21.5 bit @100 Hz sps @gain 1
Gain	1, 2, 4, 8, 16, 32, 64
Sample rate	800 (1ch-mode), 400, 200, 100, 50 sps
Input impedance	100 kOhm
Input voltage range	5–24 V

**Table 4:** Technical specifications of the microcomputer board.

CPU	1.8 Ghz quad core ARM processor and 1.4 Ghz quad core ARM processor
RAM (random access memory)	2 Gbyte LPDDR3 RAM at 933 MHz
Operating system	GNU/Linux
Storage	eMMC5.0 HS400 Flash Storage or micro SD
Power consumption	3–5 W
User interface	Tri-color RGB LED to display the status of operating system, Standard Micro-HDMI, supports up to 1920 × 1080 resolution
IO Ports, Possible expansions	30Pin : GPIO/IRQ/SPI/ADC
Connectivity	USB 3.0 Host × 2, USB 2.0 Host × 1, Ethernet RJ-45
MEMS sensor	±2 g/±6 g user selectable full-scale, Acceleration noise density (Vdd = 3.3 V; Full-scale = ±2 g): 50 μg/sqrt(Hz)
